# Marginal and internal fit and fracture resistance of three‐unit provisional restorations fabricated by additive, subtractive, and conventional methods

**DOI:** 10.1002/cre2.637

**Published:** 2022-07-24

**Authors:** Mehran Falahchai, Samiye Rahimabadi, Ghazaleh Khabazkar, Yasamin Babaee Hemmati, Hamid Neshandar Asli

**Affiliations:** ^1^ Department of Prosthodontics, Dental Sciences Research Center, School of Dentistry Guilan University of Medical Sciences Rasht Iran; ^2^ Dental Sciences Research Center, School of Dentistry Guilan University of Medical Sciences Rasht Iran; ^3^ Department of Orthodontics, Dental Sciences Research Center, School of Dentistry Guilan University of Medical Sciences Rasht Iran

**Keywords:** CAD‐CAM, dental internal adaptation, dental marginal adaptation, fixed partial denture, temporary dental restorations

## Abstract

**Objectives:**

To compare the marginal and internal fit and fracture resistance of three‐unit provisional fixed dental prostheses (FDPs) fabricated by additive, subtractive, and conventional methods.

**Material and Methods:**

Eighty 3‐unit FDPs were fabricated on metal dies of the maxillary right second premolar and second molar by four different techniques (*n* = 20): The direct method by using autopolymerizing polymethyl methacrylate (PMMA), indirect method by the compression molding technique, subtractive manufacturing by using PMMA blocks, and additive manufacturing by using digital light processing technology. The adaptation of restorations at the marginal, axial, cuspal, and fossa areas was assessed by using the silicone replica technique. After thermocycling and cyclic loading, the fracture resistance was measured by a universal testing machine. Data were analyzed by a two‐way analysis of variance (ANOVA), ANOVA, and Tukey test (*α* = .05).

**Results:**

The mean gap measured in the additive group was lower than that in all other groups at all points (*p* < .05); however, the difference in the marginal gap with the subtractive group was not significant (*p* = .995). The mean marginal and axial gaps in the subtractive group were significantly lower than the corresponding values in both conventional groups (*p* < .05). A significant difference existed between all groups regarding the mean cuspal and fossa gaps (*p* < .05). The mean fracture resistance of the additive group was significantly higher than that of indirect (*p* = .018) and direct (*p* < .001) groups, and the fracture resistance of the subtractive group was significantly higher than that of the direct group (*p* = .020).

**Conclusion:**

The digitally fabricated provisional FDPs showed superior marginal and internal fit and higher fracture resistance than the conventionally fabricated FDPs. Between the digital methods, the additive technique yielded superior internal fit.

## 1 INTRODUCTION

Fabrication of provisional restoration is an important step in the process of fixed prosthodontic treatment. Provisional restorations are used after tooth preparation until the delivery and cementation of the final restoration (Lee et al., 2017). Provisional restorations are fabricated aiming to replace the lost tooth structure in the process of fabrication of final restoration for biological and mechanical protection of the prepared tooth and stabilizing its position (Peng et al., 2020). They also allow the surrounding soft tissue to heal, play an important role as a guide for soft tissue formation, and preserve the soft tissue esthetics and health (J.‐Y. Park, Lee, et al., 2016; Raigrodski, 2015). Provisional restorations protect the prepared teeth and the gingival margin against oral bacteria and external forces (Miura et al., 2019). Moreover, they can be of great help in obviating the esthetic needs and checking the occlusion and speech before delivery of the final prosthetic restoration (Peng et al., 2020).

Internal and marginal fit play a fundamental role in the long‐term success of any type of restoration (Abduo et al., 2010; Falahchai et al., 2021; Kokubo et al., 2005; Nakamura et al., 2000; Sakrana, 2013). Optimal marginal fit preserves the periodontal health and minimizes cement degradation while optimal internal fit provides an excellent and uniform cement space without interfering with the retentive and resistant form at the time of cementation (Abduo et al., 2010; Falahchai et al., 2020; Peng et al., 2020; Sakrana, 2013). In fact, the marginal fit of provisional restorations should be as accurate as that of final restorations in order not to induce pulpal and periodontal inflammation and guarantee a successful outcome (Dureja et al., 2018). According to Mclean and von Fraunhofer, a misfit <120 µm is clinically acceptable (McLean, 1971), and the majority of researchers have agreed on this value.

Fracture is the most common cause of failure of provisional restorations (Dureja et al., 2018). Although restorations should be designed in such a way to have adequate strength, fracture still occurs. Thus, restorations should preferably have adequately high strength in order not to necessitate additional visits due to fracture, which would lead to patient dissatisfaction (Reeponmaha et al., 2020). A fracture may also occur in the process of normal masticatory function, especially in patients with dental bridges (Dureja et al., 2018).

The computer‐aided design/computer‐aided manufacturing (CAD‐CAM) technology can be divided into two categories of subtractive and additive manufacturing techniques. The subtractive manufacturing technique or milling is based on the milling of a block for the fabrication of the designed object. This technique can minimize the risk of errors in the fabrication of dental restorations since it uses prepolymerized blocks. According to some authors, provisional restorations fabricated by the CAD‐CAM technology are more favorable (Abdullah et al., 2016; Rayyan et al., 2015) and more reliable (Sannino et al., 2014) than those fabricated by the conventional methods. They also have an easier fabrication process and optimal clinical performance (Nejatidanesh et al., 2015; Schweyen et al., 2018). Nonetheless, some others have pointed to nonuniform internal surfaces, especially in the occlusal and axial areas (Abdullah et al., 2016; P. Coelho et al., 2009). Also, material waste, poor reproducibility in concave areas that depends on the diameter of the cutting instrument, and the vibration generated in the process of milling and its adverse effect on the accuracy are among the drawbacks of this method (Digholkar et al., 2016; Kang et al., 2018; Lee et al., 2017). The additive manufacturing process (three‐dimensional [3D] printing) is a fabrication method of objects by consecutive (layer‐by‐layer) addition of powder and liquid; thus, there would be no production waste (Digholkar et al., 2016; Lee et al., 2017). The digital light processing (DLP) technique is a method of polymer printing for application in digital dentistry. This technique consecutively exposes the liquid photopolymerized monomer layers to ultraviolet light to induce layer‐by‐layer polymerization and fabrication of the desired object according to the CAD data (Osman et al., 2017; Peng et al., 2020). The DLP system uses a micromirror device to polymerize each layer over the other (Mitteramskogler et al., 2014; Peng et al., 2020).

Several studies have assessed the effect of different fabrication methods of single‐unit provisional restorations, such as the conventional technique and CAD‐CAM milling, and less commonly 3D printing, on the adaptation and fracture resistance of restorations, reporting controversial results (Digholkar et al., 2016; Lee et al., 2017; Mai et al., 2017; S.‐M. Park et al., 2020; Peng et al., 2020; Rayyan et al., 2015; Reeponmaha et al., 2020; Shaikh et al., 2020; Suralik et al., 2020). Nonetheless, information regarding the adaptation and strength of multiunit provisional restorations fabricated by different manufacturing techniques is limited, and the available studies have only addressed the strength of such restorations (Alt et al., 2011; C. Coelho et al., 2021; Nold et al., 2021; S.‐M. Park et al., 2019, 2020; Sadid‐Zadeh et al., 2021; Stawarczyk et al., 2012; Suralik et al., 2020). Thus, this study aimed to compare the marginal and internal fit, and fracture resistance of three‐unit provisional fixed dental prostheses (FDPs) fabricated by the two conventional methods, additive, and subtractive manufacturing techniques. The null hypothesis was that the fracture resistance and internal and marginal fit of three‐unit provisional FDPs would not be influenced by their fabrication method.

## 2 MATERIALS AND METHODS

This in vitro study evaluated 80 FDPs fabricated by one of the following four techniques (*n* = 20): direct method by using autopolymerizing polymethyl methacrylate (PMMA), indirect method by the compression molding technique with autopolymerizing PMMA, subtractive manufacturing using PMMA blocks, and additive manufacturing using methacrylate oligomers.

**Figure 1:**
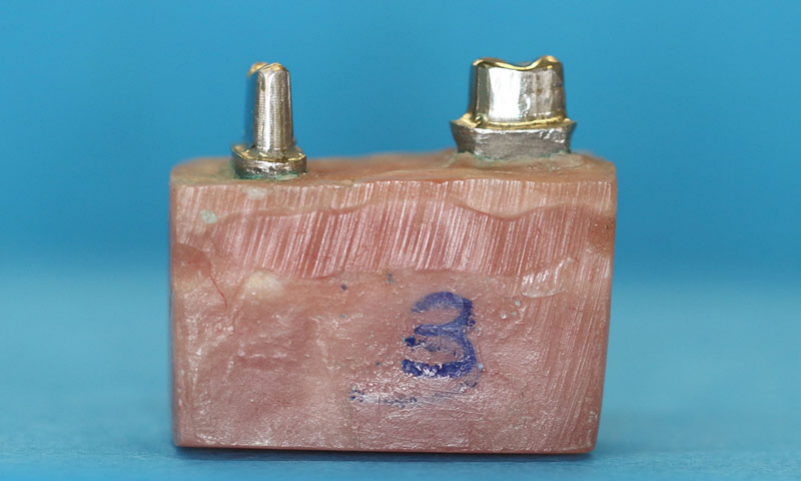
Metal die mounted in autopolymerizing acrylic resin

A total of 160 metal dies (80 s premolars and 80 s molars, Damcast NP; Damcast Dentalloy Corporation) were fabricated by the lost wax casting technique from a maxillary right second premolar and second molar with no restoration or caries that had been prepared for a three‐unit all‐ceramic bridge (Sadighpour et al., 2021). The teeth were prepared by a tapered flat‐end diamond bur (ISO 856.014; Drendel + Zweiling Diamant GmbH) and high‐speed hand‐piece under water coolant by an experienced clinician. The preparations included 1 mm of axial reduction, 2 mm of occlusal reduction, 1 mm‐thick deep chamfer finish line, and 4° taper. The two metal dies were first mounted in an autopolymerizing acrylic resin block up to 1 mm below their cementoenamel junction using a dental surveyor to simulate two teeth and an edentulous space between them (with 11 mm width corresponding to the approximate mesiodistal width of a maxillary first molar) (Figure 1). An index was made from the occlusal surface of the mounted dies by using zinc oxide eugenol and a wooden tongue blade. This index was then used for mounting other dies (Sadighpour et al., 2021).

An impression was made from the mounted metal dies with polyvinyl siloxane in addition to silicone impression material with putty and extralight consistencies (Panasil; Kettenbach) by the two‐stage technique with a special tray according to the manufacturer's instructions. The impressions were poured with type IV dental stone (GC Fujirock EP; GC Europe) with a 100 g/20 ml ratio according to the manufacturer's instructions. After 35 min, the cast was removed from the impression to fabricate the working die.

**Figure 2:**
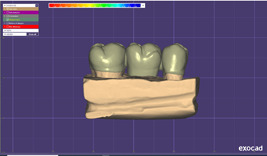
A three‐unit provisional restoration designed by a designing software

**Figure 3:**
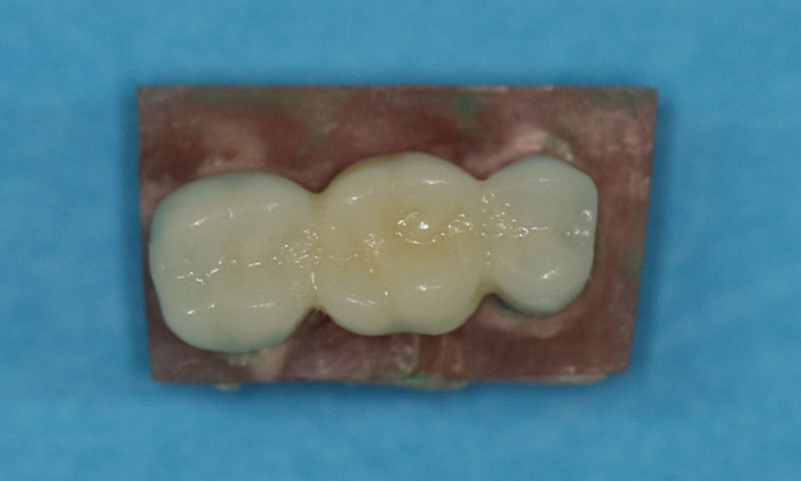
A three‐unit provisional restoration fabricated by the subtractive manufacturing technique

In the subtractive group, the working dies were sprayed with the scan spray (Dentaco GmbH & Co.KG) and scanned by a laboratory scanner (CERAMILL MAP 400; Amann Girbach). Three‐unit restorations were then designed by the CAD software (Exocad version 2020; Exocad GmbH) (Figure 2). After standardization and confirming the restoration design, PMMA resin blocks (Ceramill TEMP light 71 L20 nm; Amanngirrba AG) were milled by a milling machine (Ceramill; Amann Girrbach) (Figure 3).

In the additive group, the same restoration design file obtained by the subtractive manufacturing technique was used for the fabrication of FDPs from 3D‐printed methacrylate oligomer resin (NextDent C&B, Vertex Dental) by a printer (MAX UV; Asiga) with the DLP technology. All printed provisional restorations were cleaned with 90% ethanol for 5 min and air‐dried before polymerization. The postpolymerization process was performed in a UV photopolymerization machine (LC‐3DPrint Box; NextDent) for 30min. The parameters used for restoration design, such as the restoration dimensions (2 mm in the occlusal, 1 mm in the axial, 4 × 4 mm connector dimensions) and 60 µm cement space, were the same in both manufacturing techniques (Peng et al., 2020).

In the indirect group, a polyvinyl siloxane matrix was prepared from an FDP fabricated by the subtractive manufacturing technique. Die spacer (Die Spacer blue; Yeti Dentalprodukte GmbH) was applied on the working die 1 mm above the finish line to create a cement space equal to 60 µm (Mai et al., 2017). Provisional restorations were prepared with autopolymerizing PMMA (Tempron; GC Co.) by using the polyvinyl siloxane matrix on the working die.

In the direct group, provisional restorations were fabricated by using autopolymerizing PMMA (Tempron; GC Co.). For this purpose, a thin layer of petroleum jelly was applied to the respective metal dies. The powder and monomer were mixed according to the manufacturer's instructions. The mixture was placed in the matrix (as explained for the indirect group) in the doughy stage and the matrix was then placed on the die and remained there until the completion of polymerization. A 500‐g load cell was used to fix the restoration over the die. All fabricated restorations were inspected to ensure the absence of defects and voids. The restorations were then trimmed, finished, and polished.

**Figure 4:**
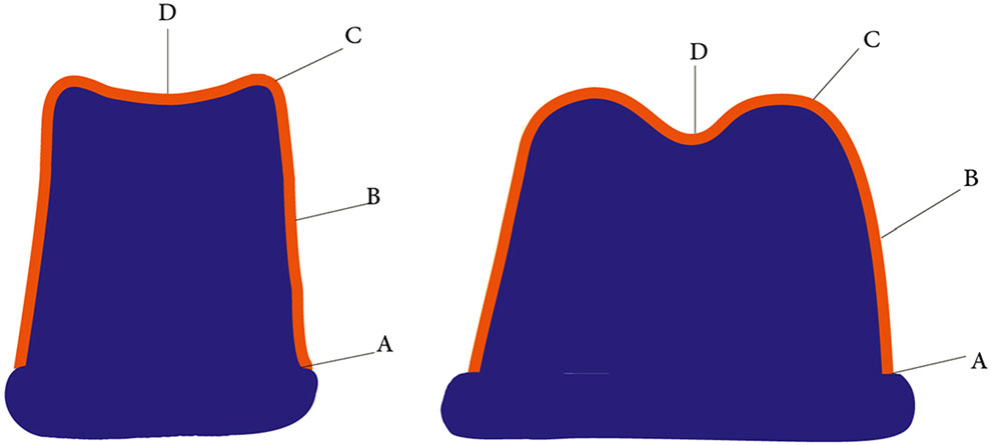
Schematic view of the silicone replica used. (A) Marginal gap, (B) axial gap, (C) cuspal gap, (D) fossa gap.

A total of 20 FDPs in each group were placed on their respective metal dies for measurement of their internal and marginal fit. The silicone replica technique was used for this purpose. Silicone material with light consistency (CharmFlex; DentKist Inc.) was applied to each crown and the restoration was placed on its respective metal die with 50 N load in a universal testing machine. After polymerization, the restoration was carefully removed from the die such that the silicone material remained in the restoration. Next, medium‐body silicone (CharmFlex; DentKist Inc.) was injected into the die containing light‐body silicone to stabilize the light‐body silicone. Next, the silicone replica was incised mesiodistally and buccolingually with a #11 surgical scalpel. By doing so, four pieces of silicone were obtained. Four points at the marginal, axial, cuspal, and fossa areas were marked on each piece for measurement under a video measuring machine (VMM, C‐Class Vision Measurement Machine; Easson Optoelectronica technology Co.) at ×202.9 magnification (Figure 4). To assess the marginal discrepancy, the absolute marginal discrepancy, which is the distance between the outermost point of the restoration margin and the external marginal line of the prepared tooth, was measured (Sakrana, 2013). To assess the internal discrepancy, the vertical distance between the internal surface of the crown and the external surface of the prepared tooth at the central points in axial, cuspal, and fossa areas was measured. All measurements were made by the same examiner who was blinded to the study objectives. The mean gap for each tooth was separately calculated for the marginal, axial, cuspal, and fossa areas by calculating the mean of measurements made in the four incised pieces. The total mean gap of each three‐unit restoration was also calculated separately for the marginal, axial, cuspal, and fossa areas by calculating the mean of the gap values of the two teeth.

The restorations were cemented on their respective metal master dies by using temporary cement (Tempbond; Kerr Italia). The cement was mixed on a mixing pad as instructed by the manufacturer. The cementation process was carried out under a 50 N load for 6 min. Excess cement was removed by a dental explorer, and the cemented restorations were stored in water for 24 h.

To simulate exposure to the oral environment for 6 months, the cemented provisional restorations underwent 50,000 thermal cycles between 5°C and 55°C with a dwell time of 60 s and a transfer time of 5 s (Reeponmaha et al., 2020). Moreover, the crowns were subjected to loading in a chewing simulator machine (SD Mechatronik). To simulate the application of masticatory forces for a 1‐month period, 20,000 cycles of 70N load were applied vertically to the occlusal surface of the restorations with 1.3 Hz frequency in distilled water at 25°C (Sadid‐Zadeh et al., 2021). All specimens were then carefully inspected under a light microscope with an LED light source at ×5.8 magnification to detect primary cracks, if any.

No crack or fracture was noted in any specimen following cyclic loading. Thus, all specimens underwent loading quasistatically. The fracture resistance of specimens was measured in a universal testing machine (Zwick/Roell Z010; Zwick) at a crosshead speed of 1 mm/min. A metal ball with a 4 mm diameter was used for loading at the center of the occlusal surface of pontic in all specimens. The mode of failure of specimens was also determined. Accordingly, specimens that fractured following load application were assigned to group F, while those that were only cracked were assigned to group C. Depending on the location of crack or fracture, the specimens were assigned to the following subgroups: Involvement of at least one connector (c), which could be associated with the involvement of pontic (cp) or at least one retainer (cr); No involvement of the connector, which could include involvement of the pontic (p) or one of the retainers (r); Involvement of the entire specimen (e).

Data were analyzed using SPSS version 24 (SPSS Inc.). The Shapiro–Wilk test was used to assess the normality of data distribution while Levene's test was applied to analyze the homogeneity of variances. Since the assumptions were met, data were analyzed using two‐way analysis of variance (ANOVA), and ANOVA, while the Tukey test was applied for pairwise comparisons at a 0.05 level of significance.

## 3 RESULTS

Table 1 presents the mean and standard deviation of marginal, axial, cuspal, and fossa gaps in provisional FDPs in the four study groups. Table 2 shows the results of two‐way ANOVA to determine the effect of fabrication method and type of tooth on the marginal, axial, cuspal, and fossa gaps. According to the results, the effect of the fabrication method on the marginal, axial, cuspal, and fossa gaps was significant (*p* < .001).

**Table 1:** Mean and standard deviation of gaps measured in micrometers

Measurement point group	Marginal gap	Axial gap	Cuspal gap	Fossa gap
Direct	143.7 ± 14.05^a^	167.82 ± 23.9^a^	74.85 ± 15.21^a^	224.84 ± 21.29^a^
Indirect	150.95 ± 21.10^a^	164.41 ± 26.20^a^	137.92 ± 50.27^b^	103.37 ± 8.87^b^
Subtractive	59.24 ± 13.35^b^	65.58 ± 20.43^b^	120.08 ± 18.60^c^	133.16 ± 19.94^c^
Additive	58.34 ± 16.15^b^	85.47 ± 21.26^c^	94.13 ± 8.02^d^	77.43 ± 19.81^d^

*Note*: Values with the same letter within the same column are not statistically different based on the Tukey test at *p* < .05.

**Table 2:** Summary of the results of two‐way ANOVA regarding the effect of fabrication method (group) and type of tooth on marginal, axial, cuspal, and fossa fit (*α* = .05)

Fit	Variable	Mean square	*F*	*p* Value
Marginal	Fabrication method	104,912	460.13	<.001
	Type of tooth	4825.38	21.16	<.001
	Fabrication method × type of tooth	905.47	3.97	.009
Axial	Fabrication method	112,140	217.61	<.001
	Type of tooth	1182.49	2.29	.132
	Fabrication method × type of tooth	1149.94	2.23	.087
Cuspal	Fabrication method	31,014.4	38.92	<.001
	Type of tooth	382.51	0.48	.489
	Fabrication method × type of tooth	697.66	0.876	.455
Fossa	Fabrication method	165,197	546.38	<.001
	Type of tooth	1789.88	5.92	.016
	Fabrication method × type of tooth	1269.85	4.2	.007

Abbreviation: ANOVA, analysis of varience.

Among the study groups, the maximum (150.95 ± 21.10 µm) and minimum (58.34 ± 16.15 µm) marginal gaps were noted in the indirect and additive groups, respectively. A comparison of the fabrication methods revealed that the marginal gap in the subtractive and additive groups was significantly lower than that in the direct and indirect groups (*p* < .001).

The maximum (167.82 ± 23.90 µm) and minimum (65.58 ± 20.43 µm) axial gaps were noted in the direct and subtractive groups, respectively. A comparison of the fabrication methods revealed that the axial gap in the subtractive and additive groups was significantly lower than that in the direct and indirect groups (*p* < .001). Also, the axial gap in the subtractive group was significantly lower than that in the additive group (*p* < .001).

The maximum (137.92 ± 50.27 µm) and minimum (74.85 ± 15.21 µm) cuspal gaps were noted in the indirect and direct groups, respectively. Pairwise comparisons revealed significant differences between all groups in this respect (*p* < .05).

The maximum (224.84 ± 21.29 µm) and minimum (77.43 ± 19.81 µm) fossa gaps were noted in the direct and additive groups, respectively. Pairwise comparisons revealed significant differences between all groups in this respect (*p* < .001).

Table 3 presents the mean and standard deviation of fracture resistance in the study groups. The maximum (726.65 ± 134.19 N) and minimum (493.22 ± 151.72 N) fracture resistance values were noted in the additive and direct groups, respectively. ANOVA revealed significant differences in fracture resistance between the study groups (*p* < .001). Pairwise comparisons by the Tukey test revealed that the mean fracture resistance in the additive group was significantly higher than that in the indirect (*p* = .017) and direct (*p* < .001) groups. Also, the mean fracture resistance of the subtractive group was significantly higher than that of the direct group (*p* = .020). No other significant differences were noted between the groups in terms of fracture resistance (*p* > .05, Table 4).

**Table 3:** Mean and standard deviation (SD) of fracture resistance in Newtons (N) in the study groups

Group	N	Mean ± SD
Direct	20	493.22 ± 151.72
Indirect	20	568.45 ± 198.68
Subtractive	20	647.69 ± 166.52
Additive	20	726.66 ± 134.19

**Table 4:** Pairwise comparisons of the study groups regarding fracture resistance using the Tukey test (*α* = .05)

Group 1	Group 2	Mean difference	95% CI	*p* Value	*F*
Direct	Indirect	75.23025	(−61.4082, 211.8687)	.475	7.48
	Subtractive	−79.23900	(−215.8774, 57.3994)	.020	
	Additive	−158.20375	(−294.8422, −21.5653)	.000	
Indirect	Subtractive	−154.46925	(−291.1077, −17.8308	.429	
	Additive	−233.43400	(−370.0724, −96.7956)	.017	
Subtractive	Additive	−78.96475	(−215.6032, 57.6737)	.432	

Regarding the frequency of different modes of failure, the results revealed that 60% of the specimens in the subtractive group, 55% of the specimens in the additive and direct group, and 25% of the specimens in the indirect group were assigned to group F. Among all specimens, fracture or crack involving the entire specimen had the lowest frequency, followed by fracture or cracking of the retainer. Fracture or cracking of the connector along with the involvement of pontic had the highest frequency (Table 5).

**Table 5:** Frequency distribution of modes of failure in the study groups

Modes of failure group	Cc	Ccp	Ccr	Cp	Cr	Ce	Fc	Fcp	Fcr	Fp	Fr	Fe
Direct	1	5	0	3	0	0	0	8	0	1	1	1
Indirect	0	8	4	1	2	0	1	2	0	1	1	0
Subtractive	3	1	1	3	0	0	3	6	1	2	0	0
Additive	3	1	0	4	0	1	1	7	3	0	0	0

Abbreviations: C, crack; c, involvement of at least one connector; cp, involvement of at least one connector along with the involvement of pontic; cr, involvement of at least one connector along with the involvement of at least one retainer; e, involvement of the entire specimen; F, fracture; p, involvement of pontic; r, involvement of one retainer.

## 4 DISCUSSION

Satisfactory internal and marginal fit of the crowns is among the influential factors affecting the success of restorations (Abdullah et al., 2016; S.‐H. Park, Yoo, et al., 2016; Peng et al., 2020). Resistance of a crown against the masticatory forces is another fundamental factor in this respect (Abdullah et al., 2018, 2016). This study assessed the internal and marginal fit as well as the fracture resistance of three‐unit provisional FDPs fabricated by the direct technique, indirect technique, and subtractive manufacturing and additive manufacturing techniques. Significant differences were noted among the study groups in both internal and marginal fit as well as the fracture resistance of restorations. Thus, the null hypothesis of the study was rejected.

Marginal fit depends on a number of factors, such as the tooth preparation design, method of measurement, impression technique, materials used, and thermomechanical aging (Angwarawong et al., 2020; Nawafleh et al., 2013; Ryu et al., 2020; Verma et al., 2020; Wu et al., 2021). It should be noted that in the fabrication of multiunit provisional FPDs, the marginal discrepancy is greater than that of single‐unit crowns due to polymerization shrinkage occurring at the pontic region (Balkenhol et al., 2008). In the present study, the mean marginal gap of the fabricated provisional FDPs was 143.74 ± 14.05 µm in the direct, 150.95 ± 21.10 µm in the indirect, 59.24 ± 13.35 µm in the subtractive, and 58.34 ± 16.15 µm in the additive group. Considering the fact that the 120 µm marginal gap is clinically acceptable, it may be concluded that FDPs fabricated by the digital manufacturing techniques had an acceptable marginal fit while the marginal fit of FDPs fabricated by the conventional methods was not optimal. According to the present results, the mean marginal gap of FDPs fabricated by the subtractive and additive manufacturing techniques was significantly lower than that of FDPs fabricated by the conventional techniques (direct and indirect groups). Nonetheless, no significant difference existed in this respect between the subtractive and additive groups. The lower marginal fit of crowns fabricated by the conventional methods can be attributed to polymerization shrinkage (Abdullah et al., 2018, 2016), which does not occur in resin blocks used in the CAD‐CAM technology due to their complete prepolymerization before milling (Abdullah et al., 2016; Alharbi et al., 2016; Koch et al., 2016). Moreover, autopolymerized PMMA has a greater amount of unreacted monomers, higher rate of water sorption, and higher number of voids than 3D printed and CAD‐CAM‐milled PMMA blocks. Therefore, it has a higher risk of greater marginal gap and deformity following placement in an environment similar to the oral cavity (Angwarawong et al., 2020). Similarly, Chaturverdi et al. (2020) reported a better fit of provisional crowns fabricated by the 3D printing technique compared with pressure molding. Peng et al. (2020) and Mai et al. (2017) reported a superior marginal fit of the crowns fabricated by the CAD‐CAM technology and 3D printing compared with those fabricated by the direct technique using autopolymerizing resin.

In contrast to the present study, Wu et al. (2021) reported superior marginal and internal fit of conventionally fabricated provisional crowns compared with those fabricated by the CAD‐CAM technology and 3D printing. This difference in the results may be due to the use of bis‐acrylic composite in the conventional fabrication of provisional crowns, which may have superior clinical performance compared with PMMA (Wu et al., 2021). In fact, materials such as methyl methacrylate, which includes monomethacrylate have higher polymerization shrinkage than composite resins due to the lower molecular weight of monomers (Burke et al., 2005; Wassell et al., 2002).

In the present study, the minimum axial gap was recorded in crowns fabricated by the subtractive manufacturing technique. This result was in agreement with the findings of Lee et al. (2017), who reported a lower axial gap of the crowns fabricated by the CAD‐CAM technology compared with those fabricated by 3D printing. This finding may be due to the evenness of the axial surface (Lee et al., 2017).

In the present study, the cuspal and fossa gaps of FDPs fabricated by the subtractive manufacturing technique were significantly higher than the corresponding values in the crowns fabricated by the additive manufacturing technique. Similarly, Mai et al. (2017) indicated a greater occlusal discrepancy between the crowns fabricated by the CAD‐CAM technology, compared with the provisional crowns fabricated by 3D printing. It should be noted that the milling technique cannot precisely refine the rugged surfaces and sharp edges due to the limitations in size, angles, and movements of the cutting instrument. Thus, the designing software programs automatically perform the relief process according to a specific algorithm to enable milling (Mai et al., 2017). This process can compromise the optimal fit of the occlusal surface (Koch et al., 2016). However, in 3D printing, materials are applied layer‐by‐layer, and the crown is fabricated by the additive manufacturing process; thus, these adjustments are not required (Alharbi et al., 2016), and better occlusal fit may be achieved.

In the present study, the mean fracture resistance of FDPs in the additive group was significantly higher than that of crowns fabricated by the conventional methods, although the fracture resistance of digitally manufactured crowns was not significantly different. This result was in agreement with the findings of S.‐M. Park et al. (2020). However, Suralik et al. (2020) demonstrated that the fracture resistance of three‐unit provisional crowns fabricated by 3D printing was significantly higher than that of crowns fabricated by the CAD‐CAM technology and the conventional method. This difference in the results may be attributed to the different study designs and materials used.

In the present study, the fracture resistance of FDPs in the subtractive group was significantly higher than that of FDPs fabricated by the direct method using autopolymerized PMMA. This finding can be due to the complete prepolymerization of blocks used in the CAD‐CAM fabrication process before milling, resulting in the formation of a polymer network with higher bearing capacity (Suralik et al., 2020). C. Coelho et al. (2021) discussed that four‐unit provisional crowns fabricated by the CAD‐CAM technology had higher strength than the traditionally fabricated crowns. However, Sadid‐Zadeh et al. (2021) found no significant difference between three‐unit provisional crowns fabricated by the CAD‐CAM technology and the conventional method in this respect. It should be noted that they used the indirect–direct technique for the fabrication of provisional crowns. Also, they used bis‐acryl composite resin for the direct fabrication of provisional crowns. Suralik et al. (2020) found no significant difference in fracture resistance between the provisional crowns fabricated by the CAD‐CAM technology and the conventional method using autopolymerized PMMA. The controversy between their results and ours may be due to the fact that they did not perform any aging process before the fracture resistance testing.

In the present study, the provisional crowns fabricated by the additive manufacturing technique were immersed in isopropyl alcohol to eliminate the unreacted monomers, which may compromise their mechanical properties (Väyrynen et al., 2016). Moreover, the marginal and internal gaps were measured before cementation and before the aging process. It should be noted that simulation of the oral environment and occlusal forces may increase the marginal gap, particularly in the crowns fabricated by using autopolymerized resin (Angwarawong et al., 2020; Dubois et al., 1999). Moreover, some authors believe that the application of luting cement can increase marginal discrepancy (Nawafleh et al., 2013; Stappert et al., 2004).

## 5 CONCLUSION

Considering the limitations of this in vitro study, it may be concluded that additive and subtractive manufacturing techniques yield more favorable results in terms of marginal and internal fit, and fracture resistance for the fabrication of three‐unit provisional crowns compared with the conventional methods. Moreover, among the digital methods, the additive technique resulted in superior marginal fit compared with the subtractive technique.

## AUTHOR CONTRIBUTIONS

**Mehran Falahchai**: Conceptualization, methodology, approving the final version of the manuscript. **Ghazaleh Khabazkar**: Resources, investigation, visualization. **Samiye Rahimabadi**: Writing—review and editing. **Yasamin Babaee Hemmati**: Writing—original draft, supervision. **Hamid Neshandar Asli**: Project administration, supervision.

## CONFLICT OF INTEREST

The authors declare no conflict of interest.

## Data Availability

The data that support the findings of this study are available from the corresponding author upon reasonable request.
